# The Rôle of Alcohol in the Treatment of Certain Diseases

**Published:** 1906-05-05

**Authors:** J. Porter Parkinson

**Affiliations:** Physician to the London Temperance Hospital and to the North Eastern Hospital for Children


### Hospital Clinics.

### THE RÔLE OF ALCOHOL IN THE TREATMENT OF CERTAIN DISEASES.

By J. Porter Parkinson, M.D., Physician to the London Temperance Hospital and to the North Eastern Hospital for Children.

The fact that alcohol is unnecessary in health is well known, and it has been shown again and again that prolonged muscular exertion is best undertaken by those who do not consume it; the experience of the celebrated Moscow campaign showed this many years ago, and more recently the testimony of the South African War was to the same effect, that the first men to fall behind during an arduous march were those who habitually took alcoholic stimulants. Even a small quantity of alcohol has a slowing effect upon mental activity, though the sensations of the person under its influence may be that mental activity is accelerated. This has been shown by the experiments of Professor Kræpelin who thought that a small quantity of alcohol accelerated the operations of adding and subtracting and learning figures; but when he came to measure on the drum the exact period of time, he found to his astonishment that he had accomplished these mental operations more slowly than before, and therefore that alcohol had a primarily deceptive influence on the mind. He also showed that the reaction time, that is, the period of time between the reception of a stimulus and the response to it, was considerably slowed during the whole time that alcohol was in active operation in the body; in this case, however, there was a very slight shortening of the reaction-time during the first few minutes after the ingestion of the alcohol. It is, therefore, necessary in judging of the effect of this drug in health or in disease, to discount absolutely the sensations and statements of the person under its influence, and to limit ourselves entirely to objective phenomena, as the sensation of greater muscular power and mental ability usually associated with wine and spirits is only an illusion.

Again, as regards resistance to disease, it has been shown by Abbot and others that alcohol, even in small doses, has a weakening influence on the structure of an animal when it is endeavouring to resist invasion by microbes, it has a slowing effect on the amœboid movements and functions of the white corpuscles, while the red corpuscles are hindered from readily yielding up their oxygen, and so it diminishes the oxidation of the tissues. This lowered resistance of the tissues is a matter of everyday experience, as shown by the susceptibility of the intemperate to pneumonia, tuberculosis, Bright's disease, and other affections.

*Typhoid Fever.*—It is now generally accepted that the majority of cases of typhoid fever do not require any stimulants at any stage of their progress. The routine practice of administering stimulants in fevers is growing gradually less and less. In the early stages of this disease it is certainly uncalled for, and if administered is very likely to accentuate the nervous symptoms, such as the headache which is so often distressing during the first week. At a later period the symptoms which are said to call for alcohol are a weak, unsteady and too frequent pulse, and the typical symptoms of the typhoid state. To take this latter first, there is no doubt that the typhoid state is much less frequently seen now than was the case fifteen or twenty years ago, and it seems likely that this is due, partly at least, to the better treatment now in vogue, such as the efficient cleansing of the tongue and teeth, and to the more frequent use of external cold for the purpose of lowering temperature, and last, but not least, to the use of various intestinal antiseptics to combat the absorption of toxins from the bowel and so to limit, partly at least, that poisoning of the nervous system to which these symptoms are due. That alcohol appears to benefit some cases in which the typhoid state has come on cannot be denied, I merely question whether this condition may not be better treated by other methods. For example, the tepid or cold bath has frequently a rapidly beneficial effect in these cases, and where circumstances render this method inconvenient the wet pack may be employed. After such treatment I have often seen the dry tongue moisten, the pulse and respiration become less frequent, the skin become cooler and moister, and the delirium disappear, the patient falling into a natural sleep from which he awakes refreshed and obviously better.

With regard to the other symptom which is said to indicate alcohol—namely, a frequent, weak, too-compressible pulse—this is no doubt due to a combination of two causes, weak action of the heart and vaso-motor relaxation. What is the effect of alcohol upon this condition? It certainly is a vaso-dilator by its action on the vaso-motor centre, and, therefore, presumably would increase the calibre of the small vessels, but in spite of this, for a time the blood-pressure is increased by a more forcible action of the heart; hence for a time the strength seems increased, the organs have a greater supply of blood, and the amount of urine is increased. These effects are, however, merely temporary, and the heart becomes more exhausted after the stimulation than it was before, and so in a patient suffering from enteric fever in which what is desired is to improve the circulation not merely temporarily for a few hours, but for days, it seems to me that alcohol is not only not indicated, but is positively harmful. In such cases I am of the opinion that cardiac tonics such as digitalis, strychnine, and perhaps adrenalin are likely to be of more value, especially when the cardiac weakness develops slowly, though in cases of sudden collapse, when it is merely necessary to tide the patient over a few hours, alcohol is, of course, most useful, and under these circumstances it is my unfailing practice to prescribe it in fairly large doses, following up its administration by one or more of the cardiac tonics mentioned above. During the five years from 1899 to 1904 inclusive, there were admitted into the London Temperance Hospital 113 cases of enteric fever, of these 10 died, showing a mortality of 8.85 per cent. The general mortality of all the fever hospitals in London for 1902, as shown in the report of the Metropolitan Asylums Board, was 15.48 per cent. Out of our 10 deaths, 7 were from causes which could not be prevented by the administration of alcohol, such as perforation, 2; peritonitis, 1; hæmorrhage, 2; meningitis, 1; while of the 30 patients who died of failure of the heart, 1 was a case of ambulatory typhoid who took to bed on the fourteenth day of his illness, and 2 had severe bronchitis initiating the heart failure. In these 113 cases, alcohol was administered to 7, of whom all but 1 recovered.

These statistics, small though they be, tend to show that alcohol is unnecessary in by far the larger number of cases of enteric fever; the same is true even when a relapse occurs, as it did in 11 of our cases.

*Pneumonia.*—In acute pneumonia the usual cause of death is circulatory failure due to the actions of poisons on the vaso-motor centres with progressive lowering of the blood-pressure. This is a much more serious factor than direct weakness of the heart itself; but sometimes death may be due to extensive involvement with œdema of the other parts of the lungs, with engorgement and progressive weakness of the right heart.

In the former case if a daily estimation of the blood-pressure be adopted as a routine procedure, the fall of pressure is easily recognised. This can be rapidly estimated by the Riva Rocci apparatus which is so simple and takes such a short time to use that it can be employed constantly, and gives much more definite information than the estimation of the compressibility of the pulse by the finger. Œdema of the lungs and distension of the right heart is recognised by the usual physical signs, combined with the increasing dyspnœa and cyanosis.

It is not disputed by physiologists that alcohol is a vaso-dilator, but in spite of this, owing to increased action of the heart, the blood-pressure rises for a time after its administration; unfortunately these good results are but transitory and the heart is more exhausted after the stimulation than it was before, hence it is only in temporary emergencies that it is of value, such as occur at or near the crisis of pneumonia. It would seem more in accord with rational therapeutics to administer strychnine and, perhaps, digitalis, which have a vaso-constrictor action on the blood-vessels and also a more lasting tonic effect on the heart. This is becoming more recognised during the last decade than formerly, when alcohol used to be considered the sheet anchor not only when circulatory failure had set in, but to prevent its incidence.

Still even now it is the practice of many to rely upon alcohol rather than upon the other drugs mentioned, whereas I think if the Riva Rocci apparatus were more generally employed and on the earliest signs of fall of blood-pressure strychnine and digitalis were used, the results would be generally more favourable. The following are the statistics of the London Temperance Hospital during the seven years 1898 to 1904 inclusive, alcohol being administered more sparingly than at most general hospitals.

Of acute pneumonia in adults there were 166 cases, of whom 40 died, giving a percentage mortality of 24.09; in children there were 216 cases of pneumonia, of whom 35 died, the mortality, therefore, being 15.74 per cent. This is, I believe, neither much more nor much less than the statistics of other general hospitals. Out of the 40 deaths in adults, however, 12 occurred shortly after admission to hospital, the patients being admitted almost in a moribund condition, and six had a clear history of heavy drinking. Alcohol was given to 16 patients out of the 382 and of these nine recovered and seven died.

It will be seen, therefore, that though alcohol was used very sparingly, the results of treatment will compare favourably with those to whom it was given more freely.

I may incidentally mention that in some cases where alcohol might have been employed I have found musk of the greatest benefit, and my experience of this drug coincides with that of Sir Dyce Duckworth, who frequently uses it in this disease.

Dr. Burney Yeo says to give alcohol in the early stages of pneumonia to prevent cardiac failure is an error, as it causes vaso-motor paresis and so aggravates the tendency to vascular engorgement. It may cause much nervous and general depression after its first stimulatory effect has passed off. It also increases the amount of toxic substances in the blood. He advocates its use in certain adynamic cases later in the disease, and recommends that it should be given in as pure a state as possible.

*Cardiac Disease.*—In chronic valvular disease of the heart when compensation is failing and residual dilatation of the cardiac chambers occurs, the chief pathological factors we have to treat are to lower the arterial tension if too high, to restore the normal nervous pressure, and to increase the strength of the muscular walls of the heart to secure effective systolic output.

The causes of the loss of compensation in the majority of cases are impoverishment of the blood-supply of the heart through the coronary arteries, physical overstrain, or diseases of other organs.

At the commencement of treatment it is important to reduce if possible the overloading of the distended heart and venous system, which will lessen for a time the work of the heart and allow the overstrained muscle a chance of working under more favourable conditions; this in many cases is sufficient to produce a marked improvement, and in slighter losses of compensation, may be, when combined with rest, all that is required. In more severe cases, however, stimulants may be necessary, till a slowly acting tonic such as digitalis has had time to produce its effects, but there seem to me to be very few cases in which the depletory treatment is not all that is necessary during the first few days, and stimulants such as strong coffee, caffeine, and ammonia may be used with advantage in them as no stage of depression follows the stimulation of these drugs. The effect of alcohol is too often that of the spur on an over-fatigued horse, producing, certainly, a temporary acceleration, but leading to increased fatigue; a better result will be attained by lessening the load and giving what rest is possible.

Alcohol, then, is not a cardiac tonic, its place in medicine is to be administered when a rapid stimulation of the feeble cardiac muscle is required, whilst other remedial agents are getting time to exert their more permanent influence, hence, when alcohol is given in these cases it should be discontinued as soon as the stimulating effect is unnecessary.

As alcohol is a stimulant and not a tonic, it would seem to be out of place to discuss its use as a tonic during convalescence from disease, but as it is so frequently employed for that purpose a few remarks may be allowed.

The feeling of well being produced by a moderate dose of alcohol is probably to a great extent responsible for its employment, but, as I have mentioned at the beginning of this paper, this feeling is but a delusion, and the vital strength is not exalted but actually lessened, while if used in any large quantity of the resistance of the body to disease is actually lowered, as it acts as a protoplasmic depressant. The following experiments attempted to show the opposite conclusion. Hau [1] showed that the use of alcohol on patients with chronic illnesses increased the bacteriolytic power of their blood towards the colon bacillus, and Miscolo and Gervino [2] found the bodies of alcoholics neutralised the toxins of tubercle provided that the alcoholism had not reached the stage of producing destruction of tissues. Against this is the weighty clinical fact that alcoholics are specially liable to tuberculous diseases.

Such divergence between physiologists and clinicians is frequently seen; for example, Dr. Leonard Hill says that most of the oxygen inhalations given to patients are useless, since it is as necessary for the blood to get rid of $CO_2$ as it is that it should absorb oxygen; and in cases of obstructed air-way it consequently is valueless. On the contrary, clinicians, at the head of whom is the President of the Royal College of Physicians, believe in the great value of oxygen in cases of pneumonia with failing heart.

Similar differences of opinion prevail with regard to the use of alcohol, and selected facts may be gathered by faddists on either side for or against its use; but it must be certainly allowed that during convalescence alcohol is not necessary, and in cases where a craving is likely to be excited by its use it should not be employed. In fact, it may be put in the same category as morphia, chloral, cocaine and the like.

Kraepelin gives as the result of his experience that the maximum daily dose of alcohol after which no depression of mental functions is to be detected is 7.5 grammes, that is, something under two teaspoonfuls, and he considers that the daily repetition of even this small dose produces a cumulative effect, and is therefore harmful.

The requirements of convalescents are sufficient rest and mental quiet to diminish the wear and tear of body and nervous system, with a sufficiency of digestible food, and alcohol operates against all these by its disturbing effect on the nervous centres and heart.

[1] Therap. Gaz., 1903. [2] Gaz. deg. Osped., 1903.

